# *mKast* is dispensable for normal development and sexual maturation of the male European honeybee

**DOI:** 10.1038/s41598-018-30380-2

**Published:** 2018-08-21

**Authors:** Hiroki Kohno, Takeo Kubo

**Affiliations:** Department of Biological Sciences, Graduate School of Science, The University of Tokyo, Bunkyo-ku, Tokyo 113-0033 Japan

## Abstract

The European honeybee (*Apis mellifera* L.) exhibits various social behaviors. The molecular and neural mechanisms underlying these behaviors have long been explored, but causal relations between genes or neurons and behaviors remain to be elucidated because effective gene manipulation methods in the honeybee have not been available until recently. We recently established a basic technology to produce mutant honeybee drones using CRISPR/Cas9. Here we produced mutant drones using CRISPR/Cas9 targeting *mKast*, which is preferentially expressed in a certain subtype of class I Kenyon cells that comprise the mushroom bodies in the honeybee brain. By immunoblot analysis, we showed that mKast protein expression was completely lost in the mutant drone heads. In addition, during the production process of homozygous mutant workers, we demonstrated that heterozygous mutant workers could be produced by artificial insemination of wild-type queens with the sperm of mutant drones, indicating that *mKast* mutant drones were sexually mature. These results demonstrate that *mKast* is dispensable for normal development and sexual maturation in drone honeybees, and allow us to proceed with the production of homozygous mutant workers for the analysis of a particular gene by gene knockout in the future.

## Introduction

The European honeybee (*Apis mellifera* L.) exhibits various social behaviors, and has long been used as an experimental animal for the study of social behaviors^1–3^. Workers, female adults of the facultatively non-reproductive caste, exhibit a division of labor depending on their age after eclosion; younger bees (nurse bees) engage in cleaning their hive or nursing the brood inside the hive and older bees (foragers) forage for nectar and pollen outside the hive, whereas queens, female adults of the reproductive caste, engage in laying eggs^1,2^. Forager workers exhibit very good learning and memory performances and convey symbolized information of the location of a food source to their nestmates by performing the waggle dance^3^. The European honeybee genome was sequenced earlier among insects, and the neural mechanisms underlying these social behaviors have been studied extensively^4,5^.

In the honeybee brain, the mushroom bodies (MBs), a higher order center of the insect brain, comprise several subtypes of MB intrinsic neurons, termed Kenyon cells (KCs); class I large-, middle-, and small-type KCs (lKCs, mKCs, and sKCs, respectively), whose somata are localized inside the MB calyces, and class II KCs, whose somata are localized at the outer bottom surface of the MB calyces^6^. MBs are involved in learning and memory in various insect species, including the honeybee^7–11^. In addition, MBs integrate multimodal sensory information in the honeybee, as projections from distinct primary sensory centers terminate in the MB calyces^12,13^. Moreover, MBs are involved in foraging behaviors in the honeybee, as the volume of the MB calyces, which are composed of KC dendrites, increases depending on the foraging experience of the workers^14^, and neural activity in the MBs is enhanced in forager brains^15,16^.

To investigate the molecular bases underlying the regulation of social behaviors, genes preferentially expressed in a behavior- and/or area-preferential manner in the honeybee brain were identified^17–22^. The KC subtypes have distinct gene expression profiles^6^. We recently found that the number of class I KC subtypes increases from one to two and then from two to three with the behavioral evolution from solitary phytophagous lifestyle (sawfly) to solitary parasitism (parasitoid wasp), and then to nidification (Aculeate Hymenoptera, including the honeybee), suggesting that the subdivision of KC subtypes is related to behavioral evolution in hymenopteran insects^23^.

**Figure 1:**
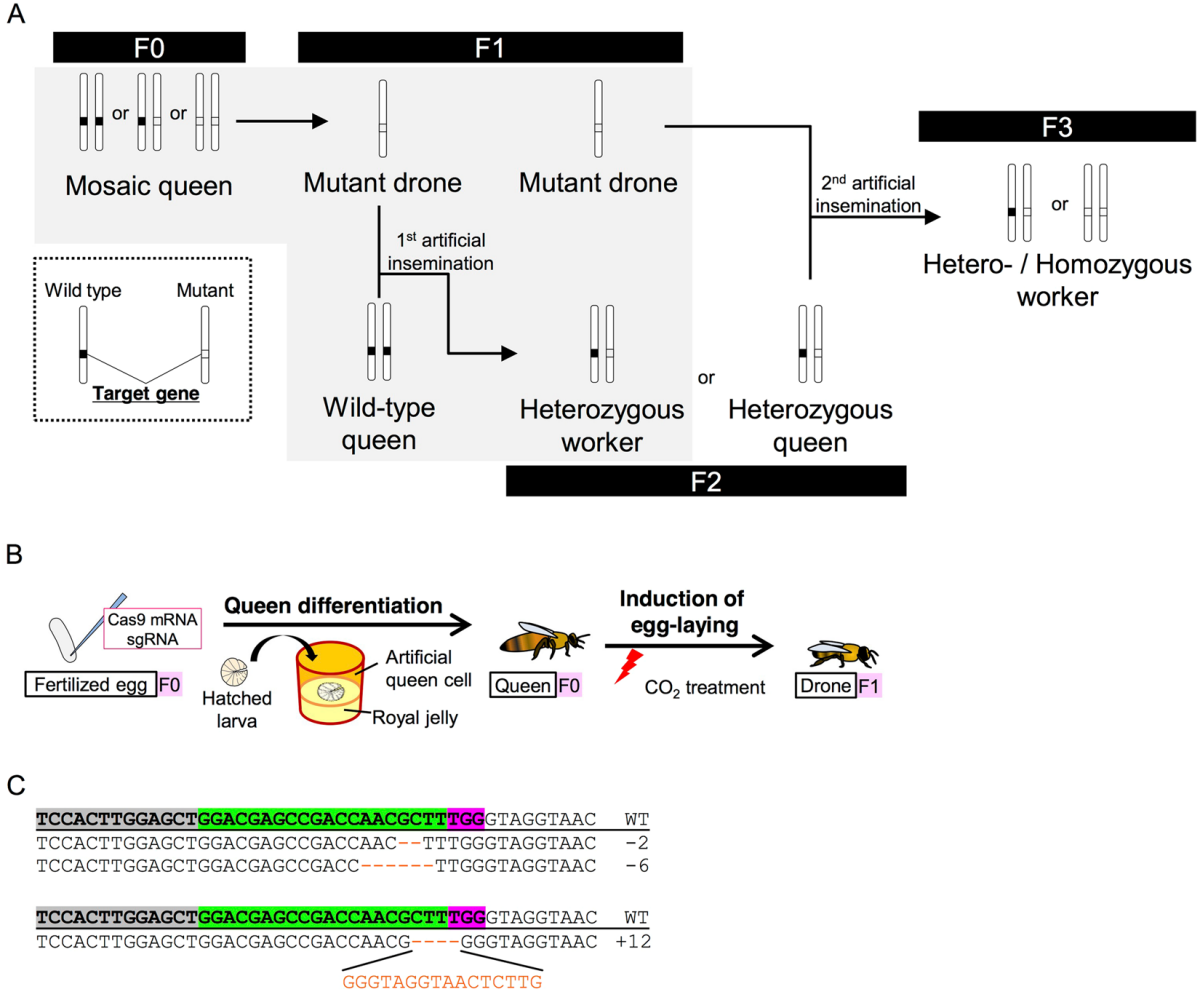
Production of *mKast* mosaic queens. (**A**) Overall procedure for the production of knockout workers. First, reproductive females with genome-edited germ cells (mosaic queen, F0) are produced. Then, wild-type queens are artificially inseminated (1st artificial insemination) with sperm from mutant drones (F1) that are derived from mosaic queens. Heterozygous female larvae (F2) derived from the wild-type queen artificially inseminated with mutant drone sperm can differentiate into either heterozygous queens (F2) or heterozygous workers (F2). Finally, heterozygous and homozygous workers (F3) are produced from heterozygous queens (F2) artificially inseminated (2nd artificial insemination) with sperm from the mutant drones (F1). White bars indicate chromosomes that have mutant (light gray boxes) or wild type (black boxes) alleles for the target gene, as shown in the lower left inset outlined with dotted lines. Note that, in the honeybee, fertilized eggs (2n) develop into females (queens or workers), whereas unfertilized eggs (n) develop into drones. Paired chromosomes thus represent female (queen or worker) individuals, whereas singlet chromosomes indicate drones. The shaded area indicates the part accomplished in this study. (**B**) Schematic diagram of the process to produce mosaic queens and mutant drones. (**C**) Indel patterns detected around the sgRNA target site in drones derived from queen No. 3 (upper sequences) and queen No. 5 (bottom sequences). The uppermost sequences with underlines are reference wild type sequences deposited in the NCBI database. Sequences with bold letters in boxes indicate exons. Green and magenta boxes indicate the sgRNA target site and PAM sequence, respectively. Deletions and insertions detected around the sgRNA target site are shown with orange dashes or letters, respectively, below the reference sequences. Numbers of total base deletions or insertion of each sequence are indicated at the right of the corresponding sequences.

The molecular and neural mechanisms underlying honeybee social behaviors, especially the functions of genes preferentially expressed in each KC subtype, however, have remained mostly unknown because effective gene manipulation methods for the honeybee were not available until recently. The first transgenic male honeybee (drone) was produced using transposon *piggyBac* in 2014, and both integration and expression of the reporter gene were confirmed^24^. We first used CRISPR/Cas9 in 2016 to produce a knockout honeybee drone that lacks *major royal jelly protein* *1* (*mrjp1*), which encodes a major protein component of royal jelly^25^. The overall procedure for the production of mutant drones and workers through the generation of mosaic queens is as follows (for detail, see also Fig. 1A): The first step is to produce queens (F0) with mutated germ cells (in our experiments, termed ‘mosaic queens’) by differentiation of the injected embryo into queens. The second step is to obligate the mosaic queens to lay unfertilized eggs that develop into drones and select mutant drones (F1) to collect mutant sperm by genotyping the drones (candidate mutant drones). The third step is to produce heterozygous mutant queens (F2) by artificial insemination of wild-type queens with the sperm from mutant drones (F1). Finally, the fourth step to produce hetero- and homo-zygotic mutant workers (F3) is a second artificial insemination of the heterozygotic mutant queens (F2) with sperm from mutant drones (F1). The previous studies went no further than producing gene-manipulated drones, however, and the subsequent processes have not yet been accomplished^24,25^. In addition, as these studies focused mainly on technologic development, there have been no detailed studies of mutated phenotypes using gene-manipulated honeybees.

Here, we conducted gene knockout using CRISPR/Cas9 methods targeting *middle-type Kenyon cell-preferential arrestin-related protein* (*mKast*), which is preferentially expressed in the mKCs in honeybee MBs^22^. *In situ* hybridization analysis revealed that *mKast* is also expressed in optic lobes (OL, a visual center), antennal lobes (AL, an olfactory center), and subesophageal ganglion (SOG, a center for regulating proboscis function)^22,26^. In addition, quantitative reverse transcription- polymerase chain reaction (PCR) analysis revealed its adult brain-specific expression^26^. These *mKast* expression patterns imply that *mKast* is related to higher-order function of the adult brain by regulating the processing of various types of sensory information. In addition, *mKast* begins to be expressed in the mKCs after the differentiation of lKCs and sKCs ceases in the pupal brain during metamorphosis, raising the possibility that *mKast* expression is associated with mKC differentiation^22,26^. To analyze the function of *mKast* in the regulation of honeybee social behaviors, it is necessary to produce *mKast* knockout workers through production of the *mKast* knockout drones (see Fig. 1A). *mKast* is also useful as a target gene because *mKast* knockout may not cause embryonic lethality, considering its adult brain-specific expression.

In the present study, we produced *mKast* mutant drones (F1) using CRISPR/Cas9, and performed immunoblot analysis to confirm the complete loss of mKast protein expression in the heads of the mutant drones. To further proceed with the production of homozygous mutant workers, we demonstrated that *mKast* mutant drones undergo normal sexual maturation, and produced heterozygous mutant workers (F2) by artificial insemination of wild-type queens with sperm from mutant drones.

## Materials and Methods

### Honeybees

The European honeybee (*Apis mellifera* L.) colonies were purchased from Rengedo beekeepers (Saga, Japan), and maintained at the University of Tokyo (Tokyo, Japan). Genome-edited honeybees must be maintained under restricted laboratory condition for legal reasons, in Japan. Therefore, colonies that contained genome-edited bees were set inside two mosquito nets (2.5 m × 2.0 m × 2.0 m) that were hung in an insectary (6.2 m × 3.6 m × 3.4 m, hereafter called the ‘flight room’) and the bees were reared at 25 °C with 40–60% relative humidity, and long-day conditions (16 h light, 8 h dark). Queenless colonies were prepared either by collecting some comb-frames that contained workers, drones, and their larvae and pupae, but not queens, from several outside colonies and placing them together in the same hives, or by removing queens from colonies. To maintain colonies in the flight room, comb-frames that contained larvae and pupae but not workers, drones, and queens, were provided from normal outside colonies whenever necessary.

### Preparation of single-guide RNA and Cas9 mRNA

The single-guide RNA (sgRNA) target site was designed in the first open-reading frame (ORF) of *mKast* (Gene ID: 725542) as far upstream as possible, and the specificity was confirmed by a BLAST search (Supplementary Fig. S1). RNA synthesis of sgRNA and Cas9 mRNA were conducted as described previously^25^. For synthesis of the sgRNA, oligo DNAs containing sequences corresponding to the sgRNA target site and restriction enzyme recognition site were synthesized (Fasmac, Japan), and annealed oligomers were ligated into the vector pDR274 (Addgene, UK) and cut with *Bsa*I-HF (New England Biolabs, Japan). After linearization using *Dra*I (Takara, Japan), the sgRNA was *in vitro*-transcribed using the T7- Flash Transcription Kit (Epicentre, USA), purified by phenol/chloroform extraction and ethanol precipitation, and stored at −80 °C until use. For the synthesis of Cas9 mRNA, the Cas9 expression vector pXT7-hCas9 (China Zebrafish Resource Center^27^) was linearized using *Xba*I (Takara, Japan), and Cas9 mRNA was *in vitro*-transcribed, capped, and poly-adenylated using an mMESSAGE mMACHINE T7 Ultra Transcription Kit (Thermo Fisher Scientific, Japan). The transcripts were purified using an RNeasy Mini Kit (Qiagen, Japan), and stored at −80 °C until use.

### Microinjection

Microinjection was performed essentially as described previously^25^, with some modifications. To collect the fertilized eggs derived from queens in outside colonies, the queens were confined in small plastic cages set inside the colonies. These plastic cages contained plastic honeycombs with a detachable bottom (Kumagaya Honeybee Farm, Japan), which facilitated the collection of eggs for microinjection. The cages were removed 3 h after they were placed in the outside colonies to collect eggs within 3 h after oviposition. sgRNA and Cas9 mRNA were diluted to 100 ng/μl each with injection buffer (10 mM HEPES, pH 6.7, containing 130 mM NaCl, 6 mM KCl, 4 mM MgCl_2_, 5 mM CaCl_2_, 25 mM glucose, and 0.16 M sucrose)^28^ and loaded into a glass capillary (Drummond, USA) whose tip was pulled and polished to a 35° angle and an outer diameter of ~5 µm. The detachable bottoms of the cages containing the fertilized eggs were fixed on an oil-based clay, and sgRNA and Cas9 mRNA were injected into the dorsoposterior part of the eggs under a stereoscopic microscope (Leica, Germany) using a microinjector (Eppendorf, Japan). The injection time was 0.2 s, the injection pressure was 700 hPa, and the balance pressure was 50 hPa. Injected eggs were incubated for about 3 days in a humid chamber containing saturated CuSO_4_ at 34 °C to maintain 98% relative humidity. To obtain an adequate number of injected larvae, we repeated the same manipulation for three successive days. Larvae that hatched approximately 3 days after injection were transferred onto artificial food (75% royal jelly, 6.4% glucose/fructose, 0.5% yeast extract, diluted in distilled water) in a Petri dish (diameter 3 cm) and maintained in the humid chamber at 34 °C. The larvae were transferred onto fresh artificial food every day until they were introduced into a queenless colony for 2 to 4 days.

### Production of mosaic queens and mutant drones

Mosaic queens and mutant drones were produced essentially as described previously^25^, with some modifications. Wild-type larvae within 3 days after hatching were collected from outside colonies, grafted onto the surface of artificial food placed in the plastic queen cells (Kumagaya Honeybee Farm, Japan), and introduced into the queenless colony placed in the flight room 1 day before introducing the injected larvae therein. The next day, the wild-type larvae contained in the plastic queen cells and fed by nurse bees in the queenless colony were replaced with injected larvae hatched 2 to 4 days before, and the cells containing injected larvae were returned to the same queenless colony. After 8 days, each queen cell completed by nurse bees in the queenless colonies was introduced into a different queenless colony to prevent newly emerged queens from killing each other and allow a single queen (candidate mosaic queen) to emerge in each colony. To induce these candidate mosaic queens to lay unfertilized eggs, they were treated with CO_2_ for 10 min at day 5 and 6 after emergence. Among the queens that laid unfertilized eggs, mosaic queens were identified by genotyping drone embryos or larvae (see details below). When adult drones began to emerge in the mosaic queen-right colony, newly emerged drones were marked on their backs with a Posca aqueous pen (Mitsubishi, Japan) once a week to identify their approximate ages. Adult drones over 14 days old were used for subsequent experiments (immunoblot analysis and artificial insemination).

### Genotyping of candidate mutant drones and heterozygous workers

Genomic DNA of drones (embryos, larvae, and adults) and workers (adults) was extracted as described previously^29^. To identify mosaic queens, drone larvae or embryos (approximately 40 individuals) derived from candidate mosaic queens were collected. Each larva or embryo was homogenized in lysis buffer (5 mM Tris-HCl, pH 8.5, containing 100 mM KCl, 2.5 mM MgCl_2_, 1%(v/v) NP40, 0.4 M sucrose, and 200 μg/ml proteinase K)^29^ in a plastic tube, and incubated at 66 °C for 2 h, followed by 92 °C for 10 min. PCR was performed to amplify the genomic region around the sgRNA target site using PrimeSTAR Max Premix (Takara, Japan) and gene-specific primers (forward: TGATGAGTTGAGAGAGGGTCG, reverse: AGTTCTGACAAACAACGCTCG). The PCR products were then treated with Exonuclease I (Wako, Japan) and Shrimp Alkaline Phosphatase (Roche), and the sequences were determined (Fasmac, Japan).

To genotype the adult drones derived from the mosaic queen and adult workers derived from a wild-type queen that was artificially inseminated with sperm from a mutant drone, a hind leg was excised from each drone or worker. The other body parts were stored at −80 °C until use. Genomic DNA extraction and PCR were conducted as described above. The PCR product amplified from the genomic DNA from each drone was mixed with half the amount of the PCR product of the wild-type drones. PCR products from heterozygous workers were expected to be heterogeneous as they were, without mixing with wild-type PCR products. The PCR products were then annealed, and treated with T7 endonuclease I (New England Biolabs, Japan) according to manufacturer’s protocol (T7EI assay). The digested PCR products were then electrophoresed in 2% agarose gels and PCR products fragments were detected by staining with ethidium bromide. PCR products with the expected band patterns on electrophoresis were sequenced as described above. Sequence data from heterozygous workers were processed using a web-based application, CRISP-ID v1.1^30^, to separate overlapping sequences around the sgRNA target site.

### Immunoblot analysis

Each adult drone head (wild-type or mutant) stored at −80 °C was homogenized in 100 μl of buffered insect saline (20 mM Tris-HCl, pH 7.4, containing 130 mM NaCl, 5 mM KCl, 1 mM CaCl_2_, and protease inhibitor cocktail; Roche, Japan), centrifuged at 10,000 g for 10 min, and then the supernatant was collected. Four microliters of the protein solution was denatured in sample buffer (50 mM Tris-HCl, pH 6.8, containing 10% glycerol, 2% SDS, 0.002% bromophenol blue, and 3% 2-mercaptethanol), and electrophoresed in polyacrylamide gel (5% for stacking, 10% for separation). Immunoblot analysis was performed essentially as described previously^26^, with some modifications, using 0.2 μg/ml anti-mKast antibody^26^ or normal guinea pig IgG (Santa Cruz Biotechnology, Germany) as the first antibody, 0.01 μg/ml goat anti-guinea pig IgG-HRP conjugated (A7289, Sigma, Japan) as the second antibody. Signals were detected using ECL select Western Blotting Detection Reagent (GE Healthcare, Japan) and ImageQuant LAS 4000mini (GE Healthcare, Japan).

For immunoblot analysis with control antibodies, the same membranes were treated with stripping buffer (62.5 mM Tris-HCl, pH 6.7, containing 2% SDS, and 100 mM 2-mercaptethanol) for 30 min at 50 °C to remove the bound antibodies. The membranes were then treated with 0.2 μg/ml anti-mouse β-actin (sc-47778, Santa Cruz Biotechnology) or mouse IgG1 negative control (Millipore, Japan), and then 0.01 μg/ml goat anti-mouse IgG (374-1806, KPL, USA). Signals were detected as described above.

### Artificial insemination

Adult drones over 14 days old that were derived from a mosaic queen were collected and the endophallus was everted by applying pressure with the fingertips to the side of the abdomen. The semen exposed at the surface of the endophallus was collected into a glass capillary backfilled with Bee Sperm Solution (20 mM HEPES, pH 8.3, containing 30 mM trehalose, 55 mM KCl, 25 mM NaHCO_3_, 82.6 mM trisodium-citrate, 0.02% streptomycin sulfate, and 0.01% penicillin-GK)^31^ using an insemination syringe (Schley, Germany). The glass capillary was changed after collecting the semen from five drones (semen pool) and stored in the dark at 25 °C for approximately 1 day until use. The drones from which the sperm was collected were then genotyped as described above using a hind leg collected from the drones. A semen pool that was confirmed to contain sperm from a mutant drone by genotyping was used for artificial insemination of a wild-type queen with an insemination instrument (Schley).

### Data availability

All data generated or analyzed during this study are included in this published article (and its Supplementary Information files).

## Results

### Production of mosaic queens and mutant drones

We first tried to produce candidate mosaic queens (F0). A specific sgRNA target site was designed in the first ORF of *mKast* as far upstream as possible to induce the complete loss of mKast protein function by frameshift mutation (Supplementary Fig. S1). Among 110 fertilized eggs injected with sgRNA and Cas9 mRNA, 29 larvae were hatched (26.4%). Among 29 hatched larvae, 17 larvae were alive and introduced into a queenless colony. Of the 17 larvae, 5 emerged as queens by introducing the hatched larvae into a queenless colony (queens No. 1–5). Four of the queens began to lay unfertilized eggs after two successive days of CO_2_ anesthetization (Fig. 1B and Table 1). Sequencing analysis of offspring drones (F1) from these queens (larvae from queens No. 2, 3, and 4 and embryos from queen No. 5) revealed that some drones derived from queens No. 3 and 5 had a mutation around the sgRNA target site (Fig. 1C and Table 2). When the genotypes of 40, 39, 40, and 10 offspring drones derived from queens No. 2, 3, 4, and 5, respectively, were examined, 2 (5.1%) and 1 (10.0%) mutant drones were detected from queens No. 3 and 5, respectively, whereas no mutant drones were detected among the offspring drones from queens No. 2 and 4 (Table 2). Among them, two drones derived from queen No. 3 possessed 2 and 6 bases deletions around the target site, respectively whereas one drone derived from queen No. 5 possessed 4 bases deletion and 16 bases insertion (a total of 12 bases insertion) around the target site (Fig. 1C). These results indicated that queens No. 3 and 5 were mosaic queens containing genome-edited germline cells. Moreover, it was confirmed that queen No. 3 produced drones that possessed 2 bases deletion around the target site and thus their *mKast* function was assumed to be lost. Although drone embryos were found in the colony of queen No. 5, neither wild-type nor mutant larvae were observed, and we thus concluded that *mKast* knockout was not responsible for the absence of larvae in the colony of queen No. 5. The embryogenesis of drones might have been impaired or drone larvae might have been eaten by workers soon after hatching due to improper conditions in the colony of queen No. 5. Therefore, drones derived from queen No. 3 were used in the later experiments.

**Table 1:** Summary of the process to produce mosaic queens.

Injection	Hatched larvae	Introduced into queenless colony	Emerged queens	Queens with offspring
110	29 (26.4%)	17	5 (29.4%)	4

**Table 2:** Summary of detection of indels in drone embryos or larvae derived from each queen.

Queen	Analyzed drones	Mutant drones
No. 2	40	0 (0.0%)
No. 3	39	2 (5.1%)
No. 4	40	0 (0.0%)
No. 5	10	1 (10.0%)

### Validation of the complete loss of mKast protein expression in mutant drone heads

**Figure 2:**
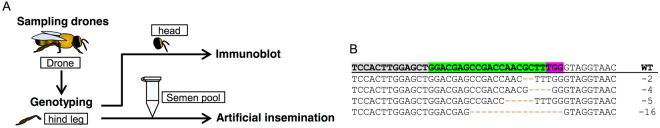
Overview of experiments using mutant adult drones. (**A**) Flowchart of experiments using mutant adult drones derived from mosaic queen No. 3. Sampled drones were first genotyped using genomic DNA extracted from their hind legs, and then, their heads were used for immunoblot analysis, and semen was used for artificial insemination. (**B**) Indel patterns in mutant adult drones used for the experiments. The uppermost sequence is the reference wild-type sequence deposited in the NCBI database. Sequences with bold capitals indicate exons. Green and magenta boxes indicate sgRNA target site and PAM sequence, respectively. Sequences around the indels detected in the analyzed drones are shown below. Detected deletions are indicated as orange dashes, and numbers of total base deletions of each sequence are indicated at the right of the corresponding sequences.

As we successfully produced *mKast* mutant drone larvae (F1), we next analyzed the physiologic effects of *mKast* knockout using mutant adult drones (F1). We designed the following experiments (Fig. 2A). First, adult drones derived from the mosaic queen No. 3 over 14 days old, which were expected to be sexually mature^32^, were collected. After their semen was collected into semen pools, their bodies were stored at −80 °C until use for the immunoblot analysis. Genomic DNA extracted from a hind leg of each drone was used for genotyping using T7 endonuclease I (T7EI assay) and sequencing (for details, see Materials and Methods). The semen pool containing the sperm from mutant drones was used for subsequent artificial insemination of wild-type queens. All insertion and deletion (indel) patterns detected around the sgRNA target site in the mutant adult drones are shown in Fig. 2B. As the numbers of bases deleted in the drones used for immunoblot analysis were not multiples of 3 (−16, −4, and −2 bp; Fig. 2), a frameshift mutation was thought to occur in these drones. Among 182 drones (larvae and adults) genotyped so far (not all drones from the mosaic queen No.3), 8 drones possessed a mutation at the sgRNA target site, and thus the proportion of genome-edited germline cells in the mosaic queen No.3 was estimated to be about 4.4%.

**Figure 3:**
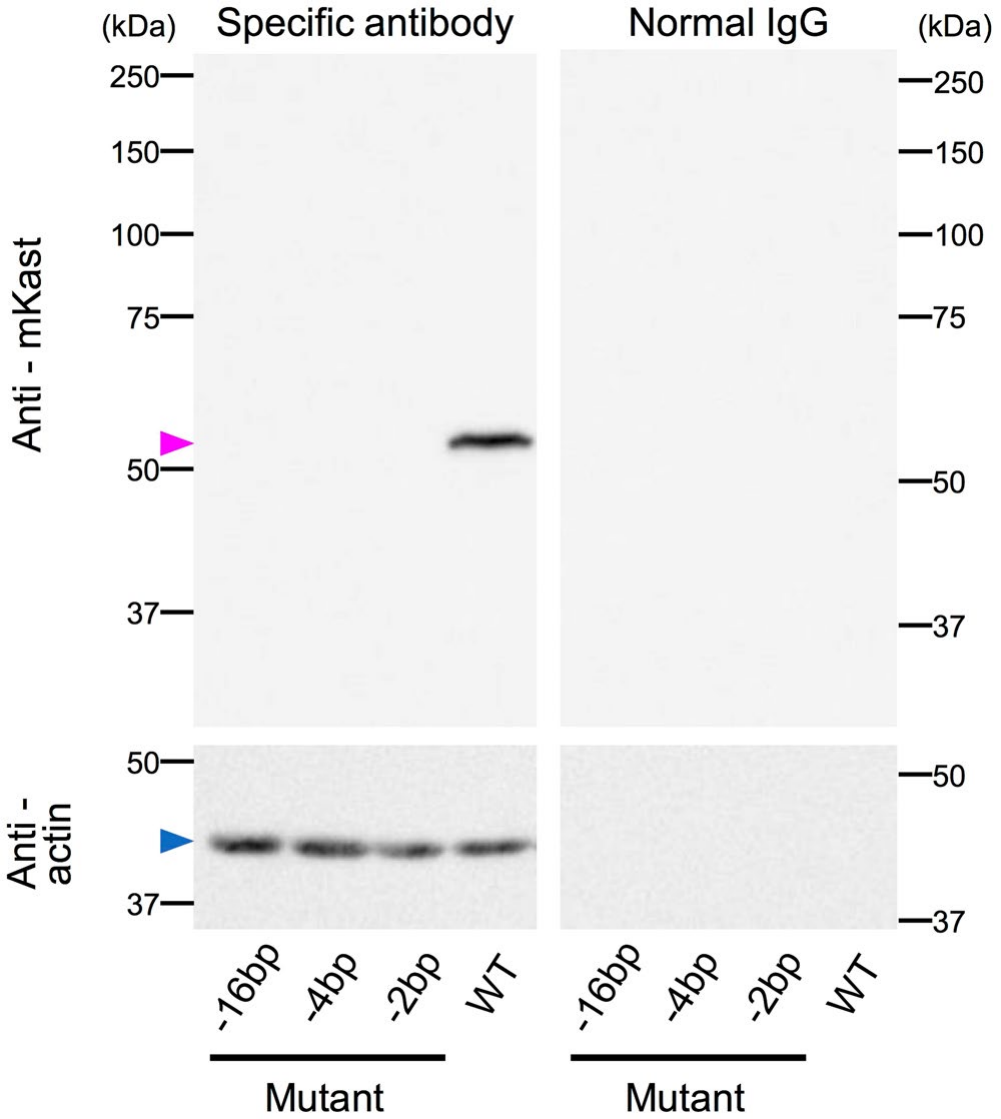
Immunoblot analysis with heads of mutant drones. Head homogenates of each mutant (−16, −4, and −2 bp) and wild type drones (WT) were subjected to immunoblot analyses with anti-mKast antibody (upper left panel), anti-β-actin antibody (lower left panel), normal guinea pig IgG (upper right panel), and mouse IgG1 control (lower right panel), respectively. Positions of molecular mass markers are shown on the left and right sides in kDa. Magenta and blue arrowheads indicate the positions of expected bands of mKast and β-actin, respectively. Full-length blots are presented in Supplementary Fig. 2. Brightness and contrast were adjusted equally across the images of the membrane treated with specific antibody and control normal IgG in each experiment.

We next investigated whether mutations in the sgRNA target site actually result in frameshift mutations, and thus the complete loss of functional mKast protein expression. As we previously prepared anti-mKast antibody by immunizing guinea pigs with recombinant mKast^26^, we utilized this antibody to detect mKast expression in mutant drone heads by immunoblot analysis. A band with the expected size (~55 kDa) was detected in the lane of wild-type drones derived from the mosaic queen No. 3 when the anti-mKast antibody was used (Figs 3 and S2). On the other hand, no signals were detected in the lanes of the three mutant drones (−16, −4, and −2 bp). The bands for β-actin (~42 kDa) were detected in all lanes when anti-β-actin antibody was used, and no signals were detected when normal IgG was used as a control experiment (Figs 3 and S2). These results clearly indicate that mKast protein was completely abolished in the mutant drone heads.

### Investigation of sexual maturation of mutant drones and production of heterozygous mutant workers

To investigate the roles of *mKast* in honeybee social behaviors, homozygous mutant workers need to be created through at least two rounds of artificial insemination using semen collected from mutant drones (Fig. 1A). Therefore, we finally investigated whether the *mKast* mutant drones (F1) became sexually matured, and semen from the mutant drones could be used to produce the next generation (F2).

**Figure 4:**
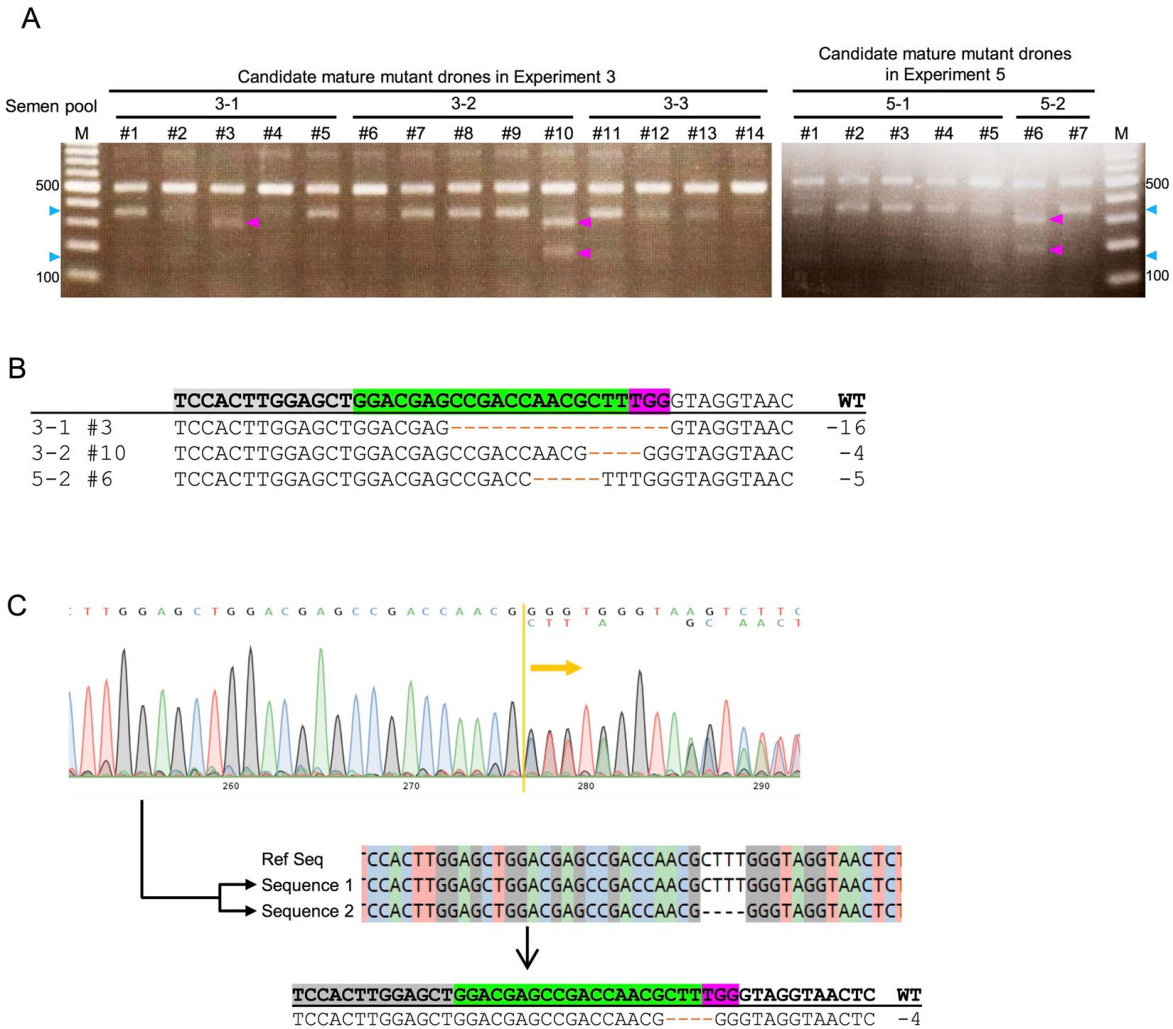
Detection of heterozygous mutant workers produced by artificial insemination. (**A**) Electrophoretic patterns of PCR products around the sgRNA target site in Experiments 3 and 5 for semen collection. Each PCR product was amplified from genomic DNA extracted from a hind leg of a sexually matured drone, and then treated with T7 endonuclease I. Magenta and blue arrowheads indicate the expected band size of the PCR products digested by T7 endonuclease I at the sgRNA target site (#3 and #10 in Experiment 3, #6 in Experiment 5) and at repeated A region, respectively. (**B**) Sequences around the sgRNA target site of mutant drones in (**A**). Orange dashes indicate detected deletions. (**C**) Overlapping sequences, which were obtained from direct sequencing of putative heterozygous mutant workers inferred from analysis using T7 endonuclease I, were separated using CRISP-ID. Heterozygous workers were derived from a wild-type queen artificially inseminated with semen pool 2 (containing semen of drone #10). Overlapping waveforms are seen at the right of the yellow bar in the upper panel. Separated sequences are shown below.

The experiments to collect semen from candidate mutant drones are summarized in Table 3. We sampled a total of 343 adult drones, and found that 33 among them were sexually mature and their semen was collected. We then conducted T7EI assay using genomic DNA of these 33 candidate mature mutant drones. The PCR product around the sgRNA target site (~500 bp) amplified from the genomic DNA of each drone was mixed with that of wild-type drones and reannealed. This manipulation is expected to result in heterogeneity (mismatch) of the PCR product at the sgRNA target site if the former drone was a mutant. As T7 endonuclease I cleaves hetero-duplex double-stranded DNA, the existence of a mismatch at the sgRNA target site of reannealed PCR products could be detected based on the band patterns on electrophoresis. Of the 33 mature drones, 3 (9.1%) exhibited the expected band patterns on electrophoresis (approximately 300 and 200 bp, #3 and #10 in Experiment 3, #6 in Experiment 5; Fig. 4A), which suggested that these drones possessed mutation around the sgRNA target site. On the other hand, two extra bands were detected in all lanes for digested PCR products (approximately 350 and 150 bp, e.g. blue arrowheads in Fig. 4A). These bands were possibly due to unexpected T7 endonuclease I digestion caused by PCR errors at the repeated adenine (A) region approximately 30–50 bp downstream of the sgRNA target site (see details in Supplementary Fig. S3). The following sequencing identified indel patterns at the sgRNA target site of these three mutant drones (Fig. 4B). Wild-type queens were then artificially inseminated with semen pools containing sperm from these mutant drones (semen pool 3-1, 3-2, and 5-2, respectively; Fig. 4A). Each semen pool was used for the artificial insemination of a single queen. As a result, only one wild-type queen inseminated with semen pool 3-2, which contained semen from a mutant drone #10, produced female offspring. As female (worker) pupae could easily be discriminated from drone pupae based on their head shape, we sampled only female pupae derived from this queen inseminated with semen pool 3-2 for the subsequent genotyping analyses.

**Table 3:** Summary of collection of semen from candidate mutant drones derived from mosaic queen No. 3.

Experiment	Collected drones	Matured drones	Matured mutants
1	47	3 (6.4%)	0
2	69	3 (4.3%)	0
3	125	14 (11.2%)	2 (1.6%)
4	75	6 (8.0%)	0
5	27	7 (25.9%)	1 (3.7%)
Total	343	33 (9.6%)	3 (0.9%)

We extracted genomic DNAs from 27 candidate heterozygous mutant workers and their genomic fragments around the sgRNA target site were amplified by PCR. The PCR products were then subjected to the T7E1 assay. Electrophoresis of the PCR product treated with T7 endonuclease I detected the expected DNA fragmentation in 5 among 27 worker samples (18.5%), suggesting that these workers are heterozygous around the sgRNA target site. Subsequent sequencing of the PCR products revealed an overlap in the waveforms just downstream of the sgRNA target site, suggesting that the PCR products contained both wild-type and mutated sequences (Fig. 4C upper panel). When the overlapping sequences contained in the PCR produces were separated using CRISP-ID^30^, one of the two sequences around the sgRNA target site corresponded to a mutated sequence of a mutant drone #10, whose semen was used for artificial insemination (−4 bp; Fig. 4C middle and lower panels), whereas the other corresponded to that of a wild-type drone. These results demonstrated that these workers were heterozygous mutants derived from mutant drone #10. In addition, the rate of heterozygous workers among workers collected for the experiment (5/27 = 18.5%) was almost comparable to that of the rate of sperm derived from mutant drone among sperm in semen pool No. 2 (~20%). These results indicated that semen from the *mKast* mutant drones is as capable as semen from wild-type drones for producing offspring and thus *mKast* is dispensable for normal sexual maturation in drones.

## Discussion

To our knowledge, this is the first report of sexual maturation of gene knockout honeybee drones and the production of heterozygous mutant workers. In addition, we successfully and reproducibly created knockout drones according to our previous reports to create knockout drones lacking *mrjp1*, which encodes a major protein component of royal jelly^25^, further confirming the efficiency of CRISPR/Cas9 for gene functional analysis in the honeybee.

In the present study, we successfully produced mosaic queens and mutant drones by genome-editing targeting *mKast*. We injected 110 fertilized eggs, and 5 of the hatched larvae successfully emerged as queens. Of these five queens, two were mosaic queens with genome-edited germline cells, which was confirmed by genotyping analysis of their offspring. The rates of larval hatching and queen emergence were almost comparable to those in our previous study (40.4% and 42.9% in^25^, 26.4% and 29.4% in this study)^25^. On the other hand, the rate of queens with offspring emerged as queens was much higher than that in the previous study (33.3% in^25^, 80% in this study). In the present study, we introduced each completed queen cell containing queen pupa into a queenless colony, whereas we introduced each queen after emergence in our previous study^25^. This might have resulted in a high acceptance rate of the introduced queen by the workers in the queenless colony, which enables us to create mutant drones and perform functional analyses more efficiently.

In the honeybee, there are three class I Kenyon cell subtypes; i.e., lKCs, mKCs, and sKCs, with distinct gene expression profiles^6,17–20,22^. Among them, *mKast* is preferentially expressed in the mKCs, whereas mKast protein is localized in all class I KC subtypes, suggesting that post-transcriptional regulation is involved in *mKast* expression in the honeybee MBs^26^. mKast contains arrestin domains and belongs to the α-arrestin family, which functions in the down-regulation of membrane receptor activity^22,33–38^. α-Arrestins regulate the function of membrane receptors such as G-protein coupled receptors, either sequentially or coordinately with β-arrestin in mammals^34,35,37^. α-Arrestin might also be related to the regulation of the Notch receptor, which regulates neural cell fate determination^36,38^. As *mKast* expression arises in the brains of late pupal stages during metamorphosis^22,26^, and cell fate determination is often mediated through membrane receptors^39,40^, we previously postulated that *mKast* is involved in the differentiation of KC subtypes, especially mKCs^22,26^. Our immunoblot analysis confirmed that mKast protein expression is completely lost in mutant drone heads. We also investigated whether *mKast* mutant drones were sexually mature, which is necessary for producing homozygous mutant workers through artificial insemination. We successfully produced *mKast* heterozygous mutant workers from a wild-type queen inseminated with the sperm from a mutant drone. These results indicate that *mKast* expression in the brain is dispensable for normal drone development and sexual maturation, and demonstrate the feasibility of conducting functional analyses of *mKast* by producing homozygous mutant workers. It might be that *mKast* expression in the brain is related to the brain functions and/or behaviors of adult honeybees. Unfortunately, both the mosaic queen and the queen artificially inseminated with the sperm of a mutant drone died in February 2018 without passing the winter, possibly due to seasonal changes and/or an altered lifespan of queens reared in the flight room. It might be necessary in the future to store frozen mutant drone sperm to make it easier to restart the process of producing mutant workers.

In the honeybee, there are four homologous genes that encode arrestin domain-containing proteins (ARRDCs), which belong to the α-arrestin family, including mKast. The similarity of the amino acid sequences among the four honeybee ARRDCs, however, is less than 40%^22^, and thus it is unlikely that gene functional compensation by other ARRDCs occurs in mutant honeybees. In addition, conservation of *mKast* sequences is quite high (~80%) among highly evolved Aculeate hymenopteran insect species, which are characterized by their nidificating behaviors, whereas conservation is much lower in parasitic wasps (~45–55%) and solitary and phytophagous sawflies (~45%, unpublished data), suggesting that unique mKast functions might have been acquired in Aculeate hymenopteran insect species^22^. Based on the *mKast* and mKast protein expression patterns, it will be interesting to investigate whether mKast is related to regulation of the same membrane receptors in primary sensory centers (OLs, ALs, and SOG) and in the higher-order center (MBs) in the honeybee brain. Recently, production of knockout mutants by genome editing was reported in several hymenopteran insect species^41–44^. The functions of odorant receptors were demonstrated to be necessary for social behaviors in ants^43,44^. The involvement of genes expressed in the higher-order center for the regulation of social behaviors, however, remains to be elucidated. We expect that functional analyses of *mKast* in the regulation of social behaviors in the honeybee could provide important clues toward elucidating the molecular and neural bases underlying honeybee social behaviors. Furthermore, comparison of the functions of *mKast* homologues with solitary hymenopteran insects, like the sawfly (*Athalia rosae*) and jewel wasp (*Nasonia vitripennis*), in which gene manipulation technologies are established^41,42^, will provide more insight into the evolution of brain functions accompanied by the evolution of genes that are related to social behaviors in hymenopteran insect species.

## Electronic supplementary material

Supplementary information
