# Supplementary material for: mKast is dispensable for normal development and sexual maturation of the male European honeybee
Source: Sci Rep. 2018 Aug 21;8:11877. doi: 10.1038/s41598-018-30380-2 (PMC6104065; doi:10.1038/s41598-018-30380-2)
Supplement: Supplementary file 1 — Supplementary information [file 41598_2018_30380_MOESM1_ESM.pdf]

Supplementary information

mKast is dispensable for normal development and sexual maturation of the male European honeybee

Hiroki Kohno[1], Takeo Kubo[1]†

1. Department of Biological Sciences, Graduate School of Science, The University of Tokyo, Bunkyo-ku, Tokyo 113-0033, Japan

†: corresponding author

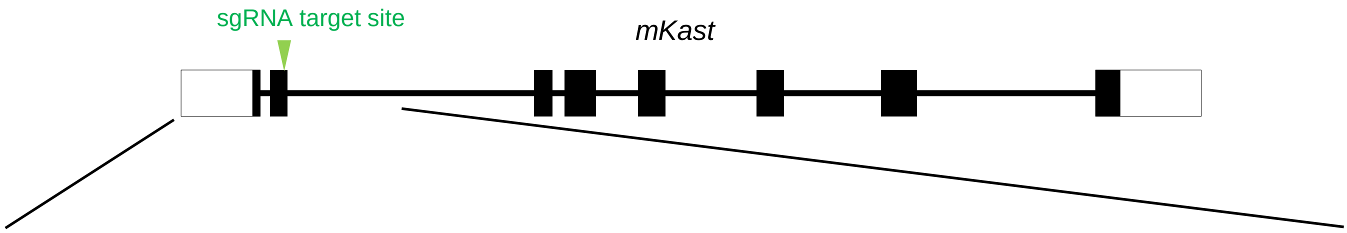

CACTGCTGCCTTAACCGGTTACCTAGTCCAACCACCCAGTCCTCCTCGCGTCCGCGCCTTTGATTTCTTTTCCCTTTTTTTTTT

Exon 1

CCCCTCCCCCCCTCCTCCCATCGATCGAAGGTTGACTGTGGCCGCGACGATATAGATCCCTCGTTCCGGGATAACTCGTTCAAC

GTCGTCATTAATCGAAAGATTTATCGGTGTATATCACGCGGCCGTGTTCGAAGAGAATGCTTATACTCGGAGAATCCGTTGTCG

AGCATCCGGTGCTCTGAATTAAGTTAAACGGGAGTGATTCTTTTCTTCGAGGAGAGAAATCCTTTTTTTTTTTTTTTTTTTGCG

AACGTGGTGAAATACATCGAGAAAGTGATAGCAAGTGCAAGAAAGTGTCTTTGAATAGAAATAGAGGAAGGAGAGGGAAGGAAG

ATTGATGAGTTGAGAGAGGGTCGTCGAAAAGGGGGTTGTACACACGCACGTGGCAGAGGATGGAGGACGAGGAGACCGAAACCG

Forward primer — Initiation codon

ATCGGTCGATCGCCGAGACTCTCTCCAAAAGGTAGAGCGAAAAAAGATTAATTAACCGTACAAGTGGGATGAGTTTCACGAATC

TGTTTCTCTGTCTGTCTCTCCGCGGTGCAGTATCGGATTCCGACGAATCGATATACTCCTCGATCACCCTCAAAAGGTCTATTA

Exon 2

CAGCGGACATCAGATCTCGGGGAACGTCCACTTGGAGCTGGACGAGCCGACCAACGCTTTGGGTAGGTAACTCTTCGAGGGTAA

sgRNA target site — PAM

TTTGAAAAAAAAAAAAGAAACGGAATTTAATCAAAAAACGATCGCGTCGAAAATAAGAAGAGAACTTGTTGTGCTACGACAGCG

TTGACTCAAACGATTCGGGGCAAGCTTTCTCCACCGTGGATATCGGAGAAAGTTGTTTCTCGACGAGCGTTGTTTGTCAGAACT

Reverse primer

GTTAAAATTTATCTGATATTCGTGAAGTAGAGATGGATATCTCTTCTTCTATATCTTTTTCGAACAGACGTCGTGATTAATCTT

CGAAAAATTTATTATTCGTCGCGAATAAATTTGTGTCGCGGAAACAAGAGTTATCGTGATACAGTTTCTCTCTCTCTCTTTCTA

GTTTTGGTAAAAGTTTCCTTCTCTATCTCGAAAGCTACGTCGAAGGATAGGTGGAAGATATTCGATTGGTAACGAGGCACGGAG

ATCAACCAGGATATATTTATTCTCTCTGGTGTTGTTCGCGATCGAAAAAAAAAAAAAAAATTTTTCTTCCCCGCCGGAGTCGAA

TGCGTGCCAAACGTGGCACGATCGATCGAGTTGCATAACGGGTGACCTGAAACGCCTTTGCCACGCTGAATACACGTCACACGG

GAGACGATGTACCGTGATTCTGGTATTGCAGGAACGCTTTTAGAATGGATAGAGCGGAGAAAAGAGTGTCCGCTATACGTAGAG

TAGCGCATTCACGAGGTGGACCTTTTCTAAGTTTAGAAAACGGATCGTTAAACAGTGTCTAATATGGTGTTG

Supplementary Figure S1. ***mKast*** **sequences around the sgRNA target site.**
(Upper panel) Schematic structure of *mKast*. Black boxes, white boxes and bars indicate coding regions (exons), non-coding regions (exons) and introns, respectively. (Lower panel) Nucleotide sequences of the 1st exon, 1st intron, 2nd exon and a part of 2nd intron. The position and sequences of initiation codon (blue under bar), sgRNA target site (green under bar), PAM sequence (magenta under bar) and primers used for PCR (orange under bar) are shown. Letters in light blue boxes indicate exons.

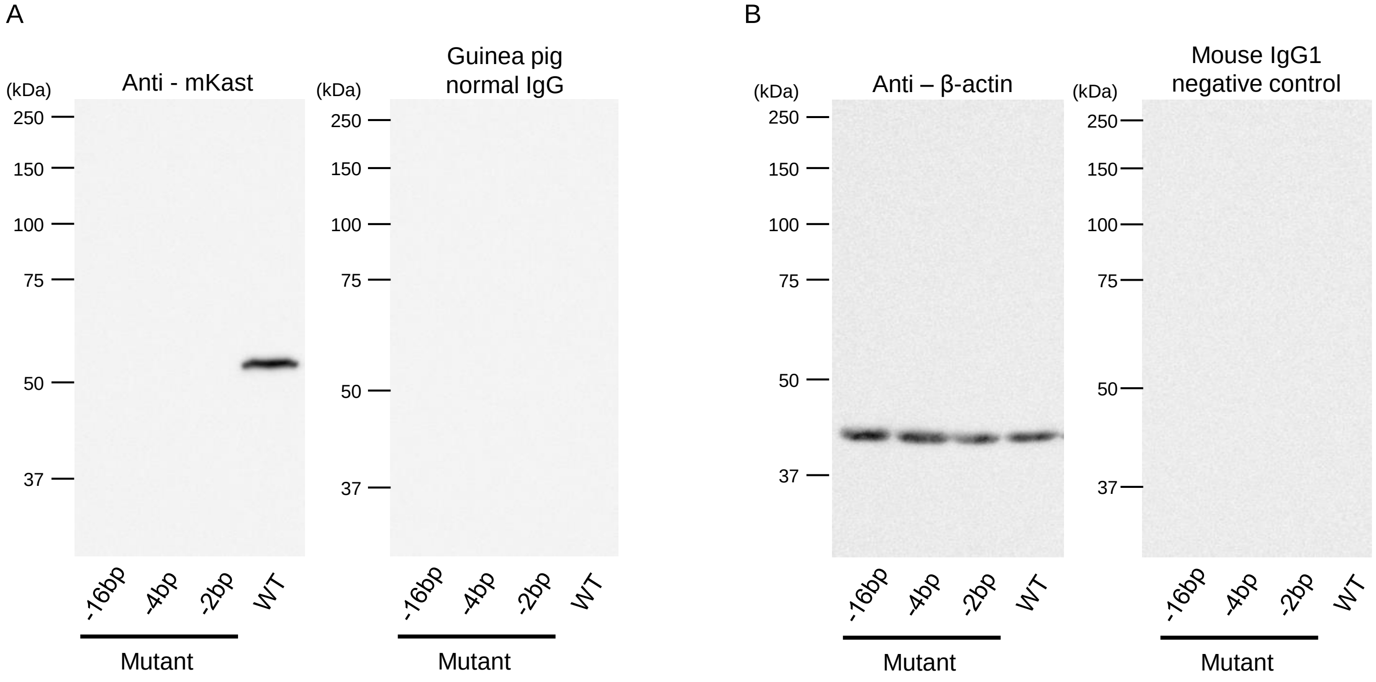

Supplementary Figure S2. The results of immunoblot analysis including full-length membranes. (A)Head homogenates of each mutant (-16, -4, and -2 bp) and wild type drones (WT) were subjected to immunoblot analyses with anti-mKast antibody (left panel) and normal guinea pig IgG (right panel). (B)The result of control experiment using anti-β-actin antibody (left panel) and mouse IgG1 control (right panel) as first antibodies. Positions of molecular mass markers are shown on the left side of each panel in kDa. Processing of brightness and contrast was applied equally across the images of the membrane treated with specific antibody and control normal IgG, in each experiment.

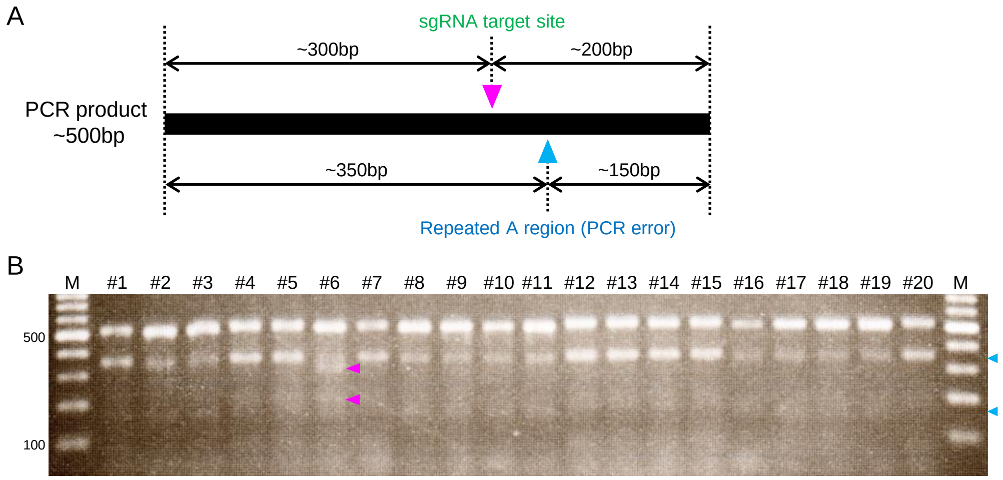

C

```
                               sgRNA target site       PAM                                   Repeated A region
Ref  TCCACTTGGAGCTGGACGAGCCGACCAACGCTTTGGGTAGGTAACTCTTCGAGGGTAATTTGAAAAAAAAAAAAG---------AAACGGAATTTAATCAAAAAA
#1   TCCACTTGGAGCTGGACGAGCCGACCAACGCTTTGGGTAGGTAACTCTTCGAGGGTAATTTGAAAAAAAAAAAAGGAAAAAAAGAAACGGAATTTAATCAAAAAA
#2   TCCACTTGGAGCTGGACGAGCCGACCAACGCTTTGGGTAGGTAACTCTTCGAGGGTAATTTGAAAAAAAAAAAAAA--------AAACGGAATTTAATCAAAAAA
#3   TCCACTTGGAGCTGGACGAGCCGACCAACGCTTTGGGTAGGTAACTCTTCGAGGGTAATTTGAAAAAAAAAAAAAA--------AAACGGAATTTAATCAAAAAA
#4   TCCACTTGGAGCTGGACGAGCCGACCAACGCTTTGGGTAGGTAACTCTTCGAGGGTAATTTGAAAAAAAAAAAAGGAAAAAAAGAAACGGAATTTAATCAAAAAA
#5   TCCACTTGGAGCTGGACGAGCCGACCAACGCTTTGGGTAGGTAACTCTTCGAGGGTAATTTGAAAAAAAAAAAAGGAAAAAAAAAAACGGAATTTAATCAAAAAA
#6   TCCACTTGGAGCTGGACGAGCCGACCAAC--TTTGGGTAGGTAACTCTTCGAGGGTAATTTGAAAAAAAAAAAAAA--------AAACGGAATTTAATCAAAAAA
#7   TCCACTTGGAGCTGGACGAGCCGACCAACGCTTTGGGTAGGTAACTCTTCGAGGGTAATTTGAAAAAAAAAAAAGGAAAAAAAGAAACGGATTTTAATCAAAAAA
#8   TCCACTTGGAGCTGGACGAGCCGACCAACGCTTTGGGTAGGTAACTCTTCGAGGGTAATTTGAAAAAAAAAAAAAA--------AAACGGAATTTAATCAAAAAA
#9   TCCACTTGGAGCTGGACGAGCCGACCAACGCTTTGGGTAGGTAACTCTTCGAGGGTAATTTGAAAAAAAAAAAAAA--------AAACGGAATTTAATCAAAAAA
#10  TCCACTTGGAGCTGGACGAGCCGACCAACGCTTTGGGTAGGTAACTCTTCGAGGGTAATTTGAAAAAAAAAAAAAA--------AAACGGAATTTAATCAAAAAA
#11  TCCACTTGGAGCTGGACGAGCCGACCAACGCTTTGGGTAGGTAACTCTTCGAGGGTAATTTGAAAAAAAAAAAAAA--------AAAGGGAATTTAATCAAAAAA
#12  TCCACTTGGAGCTGGACGAGCCGACCAACGCTTTGGGTAGGTAACTCTTCGAGGGTAATTTGAAAAAAAAAAAAGGAAAAAAAAAAACGGAATTTAATCAAAAAA
#13  TCCACTTGGAGCTGGACGAGCCGACCAACGCTTTGGGTAGGTAACTCTTCGAGGGTAATTTGAAAAAAAAAAAAGGAAAAAAAAAAACGGAATTTAATCAAAAAA
#14  TCCACTTGGAGCTGGACGAGCCGACCAACGCTTTGGGTAGGTAACTCTTCGAGGGTAATTTGAAAAAAAAAAAAGGAAAAAAAAAAACGGAATTTAATCAAAAAA
#15  TCCACTTGGAGCTGGACGAGCCGACCAACGCTTTGGGTAGGTAACTCTTCGAGGGTAATTTGAAAAAAAAAAAAGGAAAAAAAAAAACGGAATTTAATCAAAAAA
#17  TCCACTTGGAGCTGGACGAGCCGACCAACGCTTTGGGTAGGTAACTCTTCGAGGGTAATTTGAAAAAAAAAAAAAA--------AAACGGATTTTAATCAAAAAA
#18  TCCACTTGGAGCTGGACGAGCCGACCAACGCTTTGGGTAGGTAACTCTTCGAGGGTAATTTGAAAAAAAAAAAAAA--------AAACGGATTTTAATCAAAAAA
#19  TCCACTTGGAGCTGGACGAGCCGACCAACGCTTTGGGTAGGTAACTCTTCGAGGGTAATTTGAAAAAAAAAAAAAA--------AAACGGAATTTAATCAAAAAA
#20  TCCACTTGGAGCTGGACGAGCCGACCAACGCTTTGGGTAGGTAACTCTTCGAGGGTAATTTGAAAAAAAAAAAAGGAAAAAAAAAAACGGAATTTAATCAAAAAA
```

Supplementary Figure S3. Verification of the effectivity of T7EI assay for the detection of mutations at the sgRNA target site.

(A) Schematic diagram of the PCR product (horizontal black bar) and expected cleavage sites by T7 endonuclease I treatment. If mutations occur in the sgRNA target site (magenta arrowhead), T7 endonuclease I treatment results in production of ~300 bp and ~200 bp bands. On the other hand, PCR replication errors possibly due to replication slippage in the repeated A region (blue arrowhead, see Fig, S2C), result in production of ~350 bp and ~150 bp bands. (B) To examine the efficiency of the T7E1 assay, the PCR products, which were amplified from genomic DNA of drone larvae #1-20 derived from mosaic queen No. 3, were subjected to the T7E1 assay. For this, the PCR products were mixed with the corresponding PCR products of wild-type drone larva, treated with T7 endonuclease I, and then electrophoresed in a 2% agarose gel. Magenta and blue arrowheads indicate the position of band originated due to mutations in the sgRNA target site (approximately 300 and 200 bp) and PCR errors possibly due to replication slippage in the repeated A region (approximately 350 and 150 bp), respectively. M; molecular mass maker. Numbers on the left indicate nucleotide numbers of marker bands. (C) Nucleotide sequences around the sgRNA target site (green box) and PAM sequence (magenta box) of the PCR products obtained from drone larvae #1-20, which are shown in panel (B), are shown below the wild type sequence (Ref). Nucleotide sequences in the second exon are shown in gray box in the Ref sequence. Mismatch sequences in #1-20, except #16, are shown with orange dashes or letters. Two bases deletion around the sgRNA target site was detected only in drone larva #6, whereas various types of indels of A were detected at the repeated A region (blue bracket) located approximately 30-50 bp downstream of the sgRNA target site of #1-15 and 17-20. It was likely that the latter indels caused unexpected T7 endonuclease I digestion shown in panel (B), because these indels should have caused base mismatches around the repeated A region. Sequence of #16 is not shown because the sequencing failed.
